# The role of management control and integrated information systems for the resilience of SMEs

**DOI:** 10.1007/s11846-023-00657-6

**Published:** 2023-03-28

**Authors:** Paolo Roffia, Marina Dabić

**Affiliations:** 1 Department of Management, University of Verona, Verona, Italy; 2 Faculty of Economics and Business, University of Zagreb, Zagreb, Croatia

**Keywords:** COVID-19, Resilience, SMEs, Resource-based view, Dynamic capabilities, Management control, Integrated information systems

## Abstract

This paper investigates the resilience of small and medium-sized enterprises (SMEs) in relation to the COVID-19 pandemic, particularly the influence exerted by certain factors related to management control, integrated information systems (enterprise resource planning [ERP]), information and communication technology (ICT) systems, and financial resources. For this purpose, leveraging from the dynamic capability theory, in late spring 2020, a questionnaire was sent to limited-liability SMEs in Verona and Vicenza provinces in Italy operating in the manufacturing, construction, and distribution sectors. Respondents were asked to answer a set of questions and to evaluate the resilience of their firms as of January 1, 2020; May 1, 2020; and one year later, as of July 2021. Using a multivariate regression model to analyze data from the 143 questionnaires received, we found a positive influence of budgeting, business continuity tools, availability of supplementary financial resources, and resilience of the entrepreneur on both COVID-19 resilience and the organizational resilience of SMEs. SMEs with a high intention to use ERP systems also had high resilience levels. ICT use for smart working was not statistically significant. Despite the limitations concerning the sample and period considered, we believe that this study contributes to both academic debate, by illustrating the influence of certain managerial and organizational factors on the resilience of SMEs, and practice, by supporting a greater empirical use of management control tools, such as budgets and contingency plans, and integrated information systems (ERP). Our study supports the creation of financial buffers and strengthening the entrepreneur’s resilience for improving SME resilience.

**Supplementary Information:**

The online version contains supplementary material available at 10.1007/s11846-023-00657-6.

## JEL

L29, M10, M15, C29.

## Introduction

The recent COVID-19 pandemic has had a profound impact on the economic and social context of individuals (Meyer et al. 2022) as well as the operation of companies (Linnenluecke 2017; Sharma et al. 2022). Within just a few days in February 2020, Italian companies, and European ones later, had to reorganize their internal processes, maximizing their digitalization in preparation for a progressive switch-off of in-person activities (Breier et al. 2020; De Massis and Rondi 2020; Pedersen, Ritter & Di Benedetto 2020). Some companies were equipped with highly integrated information systems, such as enterprise resource planning (ERP), others had only some parts in use (legacy systems) or did not have them at all. Many companies had no business continuity plan and had never simulated their operation in such an adverse business scenario. This situation has proved particularly critical in the context of small and medium-sized enterprises (SMEs), which constitute the foundation of Italian as well as global production and tend to have limited digitalization of administrative processes, limited adoption of ERP systems, and low adoption of forecasting control tools (economic and financial budgets). Indeed, SMEs tend to be particularly vulnerable to external shocks (Eggers 2020, Kraus et al. 2013). Nonetheless, in recent decades, SMEs, which are universally recognized as having remarkable speed and agility, have also demonstrated extraordinary resilience, managing to resist and recover from crisis situations by reconfiguring their business and adopting new strategies. Whereas many studies on resilience have been conducted in large organizations, studies of smaller businesses remain inadequate (Battisti and Deakins 2017; Linnenluecke 2017; Sullivan-Taylor and Branicki 2011). These studies mostly focus on decisional processes, leadership, learning, and resource integration (Battisti and Deakins 2017; Battisti et al. 2019; Zehir and Narcıkara 2016) without addressing other important SME assets and resources, such as management control systems, integrated information systems, and digital transformation. Even the availability of additional financial resources in case of disruptive events has received little attention in the SME context, despite being recognized as an important resilience driver (Pal et al. 2014; Vogus and Sutcliffe 2007).

Will SMEs be able to withstand the crisis following the COVID-19 pandemic that has been dramatically rapid and disruptive, profoundly undermining business continuity? If so, are there specific factors related to managerial or organizational issues that can affect the resilience of the company? To answer these research questions, leveraging from dynamic capabilities theory (Helfat and Peteraf 2003; Teece 2018), we undertook a study with a survey design, using a questionnaire as a research tool. The questionnaire was sent to SMEs in the Verona and Vicenza provinces of Italy in spring 2020. Italy was the first European country to face the effects of COVID-19 on its firms, 90% of which were SMEs. To gather more public information, as well as for relevance and homogeneity purposes, manufacturing, construction, and distribution companies were selected. Chief financial officers or board of directors (BoD) members were asked about their resilience before January 1, 2020 and after May 1, 2020—the outbreak of the pandemic. We collected information regarding the use of budgets, contingency plans, ERPs, and available integrative capitals, as well as some variables related to the personal resilience of the entrepreneur, the presence of women on the BoD, the family nature of the firm, and its size. From the analysis of the 143 valid questionnaires, we found surprisingly high resilience in the SMEs and identified some managerial and organizational factors affecting this resilience.

Even with some limitations, we believe that this study contributes to the academic debate on SME resilience during the COVID-19 pandemic period in four ways. First, it highlights the importance of management control systems as a factor that promotes resilience. Second, it underlines the role of investments in integrated information systems (ERPs) and digital transformation in supporting business continuity. Third, it recognizes the significance of the availability of extra financial resources to override contingent business constraints. Fourth, it highlights the reciprocal bond between organizational resilience and entrepreneur resilience. It also has practical implications for companies and practitioners by reaffirming the importance of budget as a control tool, digital innovation in information systems (ERP implementation), and financial buffers to foster SMEs’ resilience.

## Literature review

The resilience of businesses, particularly SMEs, has been queried since the 2000s because of a series of increasingly frequent economic crises with ever stronger effects (Alberti et al. 2018; Hillmann 2021; Saad et al. 2021). Resilience is a vitally important concept in the reality of the pandemic and accompanying economic crisis, through which SMEs have had to survive. Indeed, the increased crisis frequency and disruption require an understanding of the implications or factors contributing to a reduction in their effects on companies (Amankwah-Amoah et al. 2021).

Briefly, “resilience” can be defined as the ability to resist and recover from adverse phenomena (Linnenluecke 2017; Madni and Jackson 2009). The concept has been used in various scientific fields (Hilmann 2021; Saad et al. 2021), including engineering and technical fields (i.e., the ability of systems to remain functional during and after adverse situations), the organizational field (i.e., the ability of organizations to adopt behaviors capable of reducing the impact of these adverse phenomena), the social field (i.e., the ability of individuals and communities to reduce these negative effects), and the economic field (i.e., the ability of businesses and economies to absorb negative effects caused by unexpected adverse events) (Radovic-Markovic and Tomaš 2018).

The importance of the concept of resilience in the economic field is supported by the growing attention that supranational organizations (including, recently, the International Labour Organization and the Organisation for Economic Co-operation and Development [OECD]); individual nations; and individual nations’ regional integrations, such as the European Union, are dedicating to the topic through specific reports and actions. Promoting the resilience of businesses, particularly SMEs, is now a priority and a shared objective (Ates and Bititci 2011; Dejardin et al. 2022). After all, micro, small, and medium-sized enterprises constitute the foundation of the Italian as well as the global economy, numerically representing in both cases over 90% of companies and employing more than 50% of the workforce (World Bank 2021). Of these businesses, approximately 70% are estimated to be family businesses (Zellweger 2017). In economic studies, the resilience of businesses has been studied mostly in the entrepreneurship (De Vries and Shields 2006; Korber and McNaughton 2018) and family-business literature, but some research has also focused on the organizational and managerial effects of resilience (Linnenluecke 2017; Skouloudis et al. 2020). As previously outlined, only a few studies have focused on SMEs and on the effects of managerial and organizational issues, such as management control systems, digital transformation, and integrated information system adoption (Roffia and Mola 2022), on resilience.

“Organizational resilience” was first defined as the capacity to resist and recover from traumatic events, shocks, or disasters that could affect an organization or a system either internally or externally (Coutu 2002). “Resilience” is related to the ability of a system to return to a normal state, which is different from “robustness,” which means physical strength (Annarelli and Nonino 2016). The resilience of firms has two aspects. The first concerns readiness to identify and address potential negative effects, thus is a form of *ex ante* resilience; the second is an *ex post* form of reaction to adverse effects and adaptation to new contexts (Colberg 2022; Vogus and Sutcliffe 2007). When considering the latter aspect, the resilience of a company is a resource that the company owns (or does not own) internally (Korber and McNaughton 2018). Consequently, there is a strong link between organizational resilience and the resource-based view (RBV), and the concept of dynamic capability that could be considered evolutionary (Dejardin et al. 2022). A business using the RBV approach identifies, among its sources of competitive advantage, the resources that have the traits of evaluability, uniqueness, imperfect imitability, and non-substitutability (Teece 2018). This is a relatively static view because it does not provide an explanation of how resources evolve over time in changing environments (Kraaijenbrink et al. 2010). Conversely, the concept of “dynamic capabilities” focuses on the company’s ability to renew itself and reconfigure its resources in changing environments (Dejardin et al. 2022; Teece et al. 1997; Teece 2007). These dynamic capabilities are therefore the ability of a company to create, extend, and modify its basic resources (Helfat and Peteraf 2003; Teece 2018) and are, by their nature, intrinsic and innate to the SME rather than acquired from the outside (Battisti and Deakins 2017). In line with a former study from Annarelli and Nonino (2016), Colberg (2022) proposed to split organizational resilience into two components: Operational resilience, related to organizational defenses and robustness, and strategic resilience, related to engagement in exploring opportunities. Both are linked to “organizational ambidexterity” (Raisch et al. 2009), which aims to create a competitive advantage by simultaneously exploiting existing competencies and exploring new opportunities. In the event of a crisis, operational resilience is needed for survival, as vulnerability (low) and rapidity (high) are essential for a company’s recovery after adversity (Colberg 2022), whereas strategic resilience can be used in a constant uncertain or turbulent environment without a crisis (Morais-Storz et al. 2018).

That said, in February 2020, the unexpected happened (Bouncken et al. 2022). Only a few companies had a business continuity plan in place that addressed the pandemic risk and its severe effects. Within a few days (Carugati et al. 2020; Kraus et al. 2020), Italian companies—the first in the European continent—faced an unprecedented lockdown of their physical activities. Apart from special circumstances, most SMEs in 2020 lost sales turnover by two figures (Apedo-Amah et al. 2020; Bartik et al. 2020; Caloghirou et al. 2020; Fairlie and Fossen 2022), with obvious repercussions on the value of economic capital (Scott 2002). In March, the World Health Organization declared COVID-19 a pandemic; in 2020, the virus would cause over 75,000 deaths in Italy and over 1.5 million worldwide. In addition to effecting social and health systems, the COVID-19 emergency is one of the major events that companies have had to face in recent decades (Margherita and Heikkilä 2021).

Certainly, the actions of governments, including the financial support they provided, mindful of the negative domino effects produced during the 2007–2008 financial crisis (Forbes et al. 2015), mitigated the most macroscopic effects, but the importance of being resilient to this crisis scenario inevitably emerged (Cowling et al. 2020). The COVID-19 pandemic therefore generated a new test for business resilience. The ability to resist and reconfigure by modifying, if necessary, strategies and organizational structure, by leveraging control tools, financial resources, and information systems infrastructure, has been fundamental for the survival of companies. Other positive contributions derived from the ability of a company to accelerate its digital transformation (i.e., the transition from substantially paper-based processes to digital ones), supported by integrated information systems (Muñoz-Pascual et al. 2021) such as ERP (Roffia and Mola 2022).

The choice to integrate and manage business data in a digital format is a strategic decision (Jenson and Johnson 1999) that affects all business processes, with positive influences on business innovation and entrepreneurship (Nambisan et al. 2019). However, SMEs have been traditionally rather skeptical of ERP adoption because of fears of the excessive rigidity of these systems, as well as the high cost of implementation in terms of the resources and skills required (Buonanno et al. 2005; Muscatello et al. 2003; Roffia and Mola 2022).

Nonetheless, the effects of these unforeseen adverse events can have the most serious consequences in the SME context (Ates and Bititci 2011), which is what this study seeks to address, building on the existing literature on business resilience (Bhamra et al. 2011) and analyzing the influence exercised by management control systems, integrated information systems (ERP), the availability of additional financial resources, and other company-specific variables on SME resilience. In particular, our study investigates whether the presence of economic and treasury budgets or business contingency plans, the availability of additional capital, the adoption of ERP systems and information and communication technology (ICT) for smart working, the resilience of the entrepreneur, the presence of women on the BoD, and company size affect business resilience. Regarding the potential effect of entrepreneurial resilience in the context of SMEs, which are often also family businesses, the link between the two subjects—the entrepreneur and the company—is so strong that, inevitably, a high resilience of the former has a driving effect on the latter (Ayala-Calvo and Manzano-García 2014; Branicki et al. 2018). This obviously does not exclude the positive contribution to organizational resilience provided by other individuals, such as collaborators and managers, especially in times of crisis (Ali et al. 2021; Van Der Vegt et al. 2015). Broadly speaking, a resilience of an organization is influenced by two fundamental aspects: The vulnerability of the system to which it refers (in turn an expression of its sensitivity, responsiveness, and exposure to events) and its adaptive capacity (Bhamra et al. 2011). In practice, resilience will vary from case to case, depending not only on internal variables within the organization but also on external ones. Furthermore, being more or less resilient also depends on the type of event considered. Indeed, the resilience of a given SME can be influenced by aspects related to ownership, governance, organizational size and structure, type of processes implemented, and so on.

From a theoretical point of view, the resilience of an SME derives from three “enablers”: Assets and resources, dynamic competitiveness, and learning and culture (Pal et al. 2014). Five components can be identified in the first category: Material resources (systems and management processes), finance, social systems, network relationships, and intangible assets. The second category comprises flexibility, solidity, ability to network, and redundancy. The last involves leadership style and decision-making processes, sense of community, and employee well-being.

Although scholars mostly converge on a definition of organizational resilience as the ability of an organization to reconfigure organizational resources, optimize organizational processes, reshape organizational relationships in a crisis, and recover quickly from the crisis—possibly achieving counter-trend growth—there is no universally accepted standard for the study of the structure of organizational resilience and its measurement (Chen et al. 2021). Existing studies identify two-, three-, four-, or even five-factor structures to define and measure organizational resilience, mostly identifying adaptability and organizational learning as dimensional components. In 2021, Chen et al. proposed a measurement scale for resilience based on five dimensions: Strategic resilience, capital resilience, relationship resilience, cultural resilience, and learning resilience.

However, the previous considerations on organizational resilience drawn from the literature must be placed in the context of the effects produced by the COVID-19 pandemic. As outlined in recent studies (Colberg 2022; Kraus et al. 2020; Simms et al. 2021), the pandemic has provided a unique and powerful discontinuance to internal and external environments, requiring firm adaptation in a wide set of areas. In fact, the COVID-19 pandemic has had singular connotations that have made it an exceptional event in terms of magnitude, extension, and speed (Amankwah-Amoah et al. 2021). Its effects have been dramatically disruptive and have involved the procurement, production, and distribution of goods and services in a large variety of economic sectors. Furthermore, the COVID-19 pandemic has had a never-before-seen diffusion rate (the lockdown of activities and individuals was decided and applied within a very few days), and its restrictions lasted for a long period (many months) during which new ways of working have been experimented that have leveraged on digital business spaces (Bouncken et al. 2020). In the face of these events, we can say that companies have demonstrated a great capacity for resilience, which we could define as resilience to COVID-19 more specifically than organization resilience. In fact, a few months after the outbreak of the pandemic, it was not uncommon to find companies that had previously been resilient for long time now facing increased difficulties or even failing to maintain their resilience to the COVID-19 pandemic (Amankwah-Amoah et al. 2021). Notably, traditional economic shocks have much longer forecasts and manifestation times, because they do not usually envisage a complete cessation of activities, or even of supply chains, because business activities are still possible.

The literature has offered some best practices for developing and maintaining high levels of organizational resilience (Vogus and Sutcliffe 2007) from a change-management perspective. These actions include the adoption of long-term planning measures, the strengthening of control systems, the provision of alternative scenarios, rapid decision-making and implementation of strategies, the use of performance measurement tools, the continuous collection of scenario and context information, and efficient communication (Ates and Bititci 2011).

In our opinion, in the COVID-19 pandemic context, these managerial and organizational resources, belonging to the wider set of assets and resources resilience enablers (Pal et al. 2014), will have a greater impact on the resilience of SMEs—in particular, the use of budgeting, implementation of advanced integrated information systems (ERP), adoption of ICT systems for smart working (remote communication and data sharing), and availability of supplementary financial resources to deal with treasury crises. Indeed, financial resources, along with proper budgetary control and strong financial reserves, are considered the most significant factors to keep firms buoyant amid global crisis (Pal et al. 2014).

The use of management control tools, such as economic and treasury budgets, requires the continuous monitoring of company performance through analyzing the trends of gross contribution margins and earnings before interest and taxes. It can also require the analysis of short-term cash flows and highlighting possible pressures on financial positions (Bruni 1990; Drury 2018). Accordingly, despite the growing instability and uncertainty of the past two decades (Ekholm and Wallin 2000), use of budgets supports SME resilience. The same can be said for the adoption of a corporate business continuity plan (Alesi 2008) and ERP implementation, although this practice is still not widespread in SMEs (Bhamra et al. 2011; Lindström et al. 2010). A positive support for SME resilience in times of pandemic could arise from investments in ICT (Carugati et al. 2020; Gunasekaran et al. 2011; Robertson et al. 2022), because communication interruption and information-sharing difficulty among employees were the first effects of the physical-activity lockdown evident in the first months of the pandemic. The application of supplementary financial resources (in case of need) is also judged positively in the literature (Pal et al. 2014; Vogus and Sutcliffe 2007), although this is sometimes difficult to activate in SMEs. Indeed, the COVID-19 pandemic had a dramatic effect on the cash balance of firms (Cowling et al. 2020).

Another aspect to be considered for our purposes is the family status of the company. Regardless of the difficulties in defining when a firm can be considered a family-type business, especially from an operational point of view (Roffia et al. 2021), it has already been noted that family businesses can be more resilient than non-family ones (Amann and Jaussaud 2012). According to some authors, this would be a characteristic feature because of the relationship with the family (Chrisman et al. 2011; Kachaner et al. 2012). Further socio-emotional benefits brought by family members in addition to those of an economic nature should extend the survival of businesses in evident crisis situations beyond any reasonable economic considerations, effectively increasing their resilience (Gomez-Mejia et al. 2007).

According to previous studies, organizational resilience and business performance in SMEs are often associated with managerial resilience and, particularly, entrepreneur resilience (Atiase et al. 2022; Ayala-Calvo and Manzano-García 2014; Fisher et al. 2016; Förster et al. 2022; Saad et al. 2021; Santoro et al. 2021; Sharma et al. 2022). Entrepreneurial resilience and an individual’s self-efficacy support one another to affect the behavior and decision-making of an entrepreneur, particularly in turbulent times (Castro and Zermeño 2020; Chen et al. 1998; Sharma and Rautela 2022). Also worthy of consideration are the possible influences on resilience exercised by women holding top management positions (Casprini et al. 2022; Cosentino and Paoloni 2021; Torchia et al. 2018) and company size. However, there are conflicting positions among scholars about this (Salavou et al. 2004).

This study undertook an empirical verification of the validity of certain factors in influencing the resilience of SMEs to the crisis originated by the COVID-19 pandemic to ascertain whether, eventually, they may also be valid for the general resilience of SMEs. In summary, based on the previous considerations, we formulated the following hypotheses:

H1: The adoption of management control tools, such as budgets and business continuity plans, affects the resilience of SMEs to the COVID-19 pandemic.

H2: The availability of additional financial resources, activated if needed, strengthens the resilience of SMEs to the COVID-19 pandemic.

H3: The adoption or intention to adopt ERP-type information systems and ICT tools for smart working promotes high levels of resilience to the COVID-19 pandemic in SMEs.

H4: Other factors related to the context of SMEs, such as company size, entrepreneur resilience, family-business type, or presence of woman members on the BoD, can have a significant influence on resilience to the COVID-19 pandemic.

H5: Factors possibly influencing resilience to the COVID-19 pandemic are “common” to the organizational resilience of SMEs.

## 1. Research methodology: Instruments, participants, and procedure

To answer the research questions, a quantitative study was carried out based on a questionnaire addressed to manufacturing, construction, and distribution companies with between 10 and 249 employees operating in the provinces of Verona and Vicenza in the Veneto region of Italy. According to the Italian National Institute of Statistics (Istituto Nazionale di Statistica [ISTAT]), in 2019, these two provinces were at the top levels in terms of per-capita number of firms, value of exports, and employment rates in Italy. In the same year, 5,421 limited-liability SMEs, employing around 167,000 workers, were active in this area in the manufacturing, construction, and distribution macro-sector. These firms represent a homogeneous subset of Italian SMEs suitable for research purposes (De Massis et al. 2013).

From the definition of an SME adopted within the European Union, we decided to use only the requirement regarding employees, leaving out the simultaneous use of sales revenues and total balance sheet assets. Data collection started in May 2020, three months after the start of the COVID-19 pandemic in Italy. We decided to ask SMEs about their resilience to the COVID-19 pandemic and, in broad terms, enquire about the organizational resilience of their business.

In a preliminary phase, two members of the BoD were interviewed to gather some basic information regarding our topic (Woodside and Wilson 2003), which was useful for preparing an online questionnaire through which we could ask respondents to express themselves on a Likert scale from 1 to 7 (1 = totally disagree, 7 = completely agree) on several statements relating to resilience and a set of potentially influencing factors. Respondents were asked to refer to two time points—January 1, 2020 and May 1, 2020— before the pandemic and two months after the introduction of restriction measures in Italy to mitigate the effects of COVID-19. A variable was associated with each statement, but the overall number of statements was limited to raise the response rate. A complete list of the variables considered is given in the Appendix. About 5,000 SMEs fulfilling our requirements in terms of legal form, status (in business), geographical area, and size were contacted via email from 5,421 companies listed by the ISTAT in 2019. The invitation was addressed to the chief financial officer, a member of the BoD, or the majority shareholder (depending on our contacts), because these were the most qualified individuals to discuss management control systems and integrated information systems, as well as being the main decision-makers regarding their adoption. The questionnaire indicated the purpose of the research and provided guidance on how to respond. In total, 170 questionnaires were collected, but for homogeneity with respect to the purposes and our target companies, the analysis was limited to 146 cases, three of which were subsequently removed as outliers. We addressed potential non-response bias in our sample companies, compared with SMEs active in our reference area, by comparing the responses given by the first and the last quartiles, finding nonsignificant statistical differences.

Despite the need for resilience measurement, few organizational resilience scales have been developed in the SME context (Alberti et al. 2018; Chen et al. 2021; Saad et al. 2021). In addition, given the mentioned characteristics of the COVID-19 pandemic scenario in contrast to other crisis situations, with the support of the two pilot interviews we made before the delivery of the questionnaire, we decided to focus on the core definition of resilience (the ability to resist, recover, reconfigure, and adapt strategies). In this way, we limited the variables used to assess organizational resilience, mostly testing the resilience robustness construct (Kantur and Say 2015), which we felt was critical in our context. To investigate the resilience of companies and understand its different facets, we asked four different questions to the interviewees, then summarized in one single factor (Cronbach alpha > 0.84). The survey was conducted in relation to both resilience to the COVID-19 pandemic (specific) and the organizational resilience of the company (general). The other variables we enquired about were related to the use of economic or financial budgets; the presence of a business continuity plan; the availability of additional financial resources in case of need; the resilience of the entrepreneur; the presence of women inside the BoD; and other demographic information, such as the year of birth, the NACE (Nomenclature of Economic Activities) code (the standard European nomenclature of productive economic activities), the number of employees, and the sales turnover.

Considering our literature review, budgets used as control tools and the preparation of a business continuity plan can improve the resilience of a business by forecasting financial results and simulating how to guarantee continuity of action in crisis situations. Likewise, the availability of supplementary financial resources can help to overcome the financial stress arising from the limitation of activities. Thus, we monitored the intention to use ERP-type systems and the use of ICT for smart working. Traditionally, SMEs distrust the adoption of advanced digital infrastructures, and this has postponed their implementation for many years. Hence, because of the unpredictability of contingent situations worsening, which has caught many of them in midstream, and because of the short period considered (January to May 2020), which did not allow effective changes as a result of the lockdown of many service activities, we focused on the intention to adopt management control and integrated information systems and to use ERP-type systems (rather than their actual use) (Roffia and Mola 2022). Intention is quick to change, especially when unforeseen events occur. We also invited respondents to indicate in notes any further changes that occurred between the reference date of the questionnaire (May 2020) and the date of delivery (July to September 2020).

ERP is generally known to allow for the integrated management of a company’s (digital) information system, making it possible to operate the company remotely. We decided to identify the use of a second tool category, ICT for smart working, because this also allows remote communication between different people and the exchange of documents and information. To increase the reliability of the answers, this variable was initially identified in the opposite direction (as not used) and then reversed for the subsequent analysis. We also decided to analyze the degree of resilience of the entrepreneur (in addition to that of the SME) and the degree of participation in decision-making of the BoD by woman members, as explored in other studies (Huse and Solberg 2006; Torchia et al. 2018). Finally, we included some context variables, such as the family nature of the company and its size in terms of number of employees, because of their potential influence in our SME context (Roffia, Simón-Moya, and Sendra García 2021). Regarding the first context variable, a firm was considered a family business when the family had ownership control and at least one member active on the BoD (Roffia et al. 2021). Regarding the second variable, we decided to use the natural logarithm of the number of employees as a measure of company size.

Before defining the model and carrying out the first descriptive statistics, we conducted exploratory factor analysis on the variables relating to resilience to COVID-19, general organizational resilience, control tools and business continuity plans, and the availability of additional capital. Through this step, it was possible to reduce the analysis of the aspects mentioned to four factors/variables: R2 (COVID-19 resilience), R1 (general organizational resilience), C (control tools), and BC (availability of capital). For the latter three (R1, C, and BC), identification was performed in respect of both time *t*_0_ and time *t*_1_, so the final data set encoded these factor data as R10, R11, C0, C1, BC0, and BC1.

The analysis used to answer the research questions is based on multivariate ordinary least squares (OLS) regressions, in which the dependent variable is resilience, and the explanatory variables are those previously mentioned and reported in Table 1. In turn, resilience was considered in a dual way—namely, to COVID-19 and in general terms, referring to the organizational resilience of SMEs; therefore, two separate regression models were used, one for each resilience type.

To perform a longitudinal analysis of resilience to the COVID-19 pandemic, we undertook a second delivery of the questionnaire in 2021 to the 143 respondents who had previously completed it in 2020. We named these variables R22, C2, ERPI2, ICTU2i, ICTU2, G12, and G22. The questions were asked to SMEs from July 1, 2021. As the response rate was particularly low (we collected only 46 valid responses) for 2021, we present only descriptive statistics.

## Data analysis

Table 2 shows the descriptive statistics of the variables considered in this study in our sample of SMEs, including the minimum, maximum, mean, and standard deviation.

The data referring to May 2020 (*t*_1_) in Italy, after two months of lockdown, showed a more than fair capacity of resilience declared by the companies, both to COVID-19 (R2 mean = 5.2) and in general (R11 mean = 4.9), even if resilience to the latter had been decreasing (*p* < 0.01) since the beginning of the year (R10 mean = 5.2) and had a slightly higher standard deviation (1.3 against the previous 1.2). These results are in line with what has been reported by other studies on the resilience of companies in their ability to resist and reconfigure themselves to adverse situations (Kraus et al. 2020). The 2021 data confirm and extend these results, which were even better in 2021, rising to 5.35 and 5.34, respectively.

Factor C1, which includes variables relating to the use of an economic budget, the use of the treasury budget, and the preparation of a business continuity plan, and factor BC1, which represents the availability of supplementary capital, had good mean values (4.0 and 4.1, respectively). Notably, we found the data expressed by a higher standard deviation had greater variability, 1.9 and 1.6, respectively (always on a seven-point Likert scale). Surprisingly, contrasting with the situation on January 1, 2020, the COVID-19 pandemic did not substantially change the adoption of control tools (*p* < 0.01), whereas the availability of additional financial resources decreased slightly (*p* < 0.01). In other studies on SMEs, the ability of smaller organizations to be equipped with control tools emerged (Roffia and Mola 2022), as well as the availability of supplementary finances that can be activated when needed (Bourletidis and Triantafyllopoulos 2014; Iborra et al. 2020). Data from 2021 confirm the previous trends for both control tools and financial resources (which rose to 4.413 and 4.521, respectively).

However, much more evident is the difference between January 1, 2020 and May 1, 2020 in terms of the intention to use ERP systems and the use of ICT tools for smart working. These means rose from 5.07 to 5.19, and from 3.76 to 3.97, respectively (*p* < 0.01), without changes in the standard deviation (Roffia and Mola 2022). The resilience qualities of the entrepreneur were high (mean = 5.692), as well as the presence of women on the BoD (4.566). Both grew in 2021 (to 5.804 and 4.630, respectively, p < 0.01). Approximately 70% of the companies declared that they were family firms, according to the previously defined criterion. This is not very different from data in other studies in the Italian and European contexts (Roffia et al. 2022). Company size in terms of employees fluctuated between 10 and 220 employees (ln 220 = 5.394).

Table 3 shows the correlation matrix between the variables at time *t*_1_ used in the linear regressions referred to in Table 4. The significant correlations (*p* < 0.05) show some associations between the two resiliencies R2 and R11 and the variables previously illustrated—however, without exceeding values of 0.5 (the correlation between R2 and R11 is also reported but it is not useful for our analysis, since they are alternative outcome variables in our models).

Table 4 reports the regression analyses carried out regarding resilience R2 (to COVID-19) and R11 (general) as output variables. The analysis proceeded in successive steps to insert additional explanatory variables in the model to increase its goodness of fit, expressed by the *R*^2^ value and the associated *F* test. Therefore, in Column 1, only the control variables family firm (FAM) and company size (lnEMP2019) have been inserted; Column 2 also includes the independent variables related to the resilience of the entrepreneur (G1) and the presence of women on the BoD (G2); finally, in Column 3, use of economic and treasury budgets, presence of continuity plan (C1) and availability of unused or supplementary financial resources on request (BC1), as well as ERP intention to use (ERPI) and ICT for smart working use (ICTUi) are also added into the regression. Passing from the first to the third column, a progressive increase in *R*^2^ can be found (from 0.01 to 0.35), with gradually increasing *F* test values (in the third column, the value is close to 10, *p* < 0.001). The adjusted *R*^2^ value is substantially in line with this, while modifying the number of regressors considered.

The post-regression checks verified the existence of the conditions for the validity of the model, particularly the absence of heteroskedasticity, collinearity (variance inflation factor), and the correct formulation of the model. In observing the results of Column 3, the variables found significant and, therefore, influencing SMEs’ resilience to COVID-19 were the use of management control tools (budgets and contingency plans) (C1 [+]), availability of incremental finance (BC1 [+]), intention to use ERP (ERPI1 [+]), and resilience of the entrepreneur (G1 [+]). All four variables revealed a positive influence on resilience to COVID-19, as expected. Slightly significant (*p* < 0.1), but with a negative sign, was the influence of the presence of woman members on the BoD (G2 [–]). The remaining variables were not significant except for the constant of the model. Among these was use of ICT tools for smart working (ICTU1i), which was therefore unable to affect the levels of resilience, perhaps also because by the time *t*_1_, to which the analysis refers, companies had already adopted these tools in a generalized way. Notably, actual use of ERP tools—as noted in the preliminary phase of the correlation analysis—was not significant and therefore discarded in our analyses. Columns 4 and 5 of Table 4 retrace the previous analysis focused on resilience to the COVID-19 pandemic on the organizational (general) resilience of SMEs. The results are similar to those for resilience to the effects of the COVID-19 pandemic.

## Discussion

The COVID-19 pandemic has had dramatic effects on the business operations of SMEs. The cessation of physical activities, the generalized contraction of economic activities, and the interruption of supply chains have deeply undermined the survival of enterprises, particularly SMEs, which we know are particularly vulnerable to external shocks. To increase their resilience—that is, their ability to resist and reconfigure themselves to these phenomena—is fundamental to their survival.

Few studies have specifically addressed SME resilience, and even fewer have focused on the contribution to resilience made by management control tools and integrated information systems. Moreover, the COVID-19 pandemic created an extremely serious emergency, the like of which had never occurred in the economic history of companies. Therefore, analysis of SME resilience in this new and disruptive scenario was essential.

The results from our study reveal that management control tools, such as budgets and contingency plans, support SME resilience. Companies that draw up monthly economic and treasury budgets can predict and possibly manage better situations of economic and financial tension, thereby increasing their resilience. After all, strengthening the anticipatory knowledge of negative phenomena allows SMEs to better manage their potential negative effects searching for alternatives and solutions.

Our study analyses two other aspects that, according to business operators, were fundamental to addressing the disruptive effects of the COVID-19 pandemic in 2020: Integrated information systems (ERP) and ICT tools for smart working (communication and remote information sharing). Their absence was one of Achilles’ heel of SMEs. Only in recent times, thanks to developments in technology and probably a greater awareness of their importance, have these been partially solved by investments aimed at strengthening information system digitalization and integration. Our study notes that even if intention to use ERP is associated with greater resilience, it is not yet in full maturity phase, as many more SMEs could adopt them. A little more complex is opinion on the use of ICT for smart working and its support of organizational resilience. Contrary to expectations, our data indicate the use of ICT for smart working was not statistically significant in its contribution to resilience. We interpret this result to mean that, our respondents, which included SMEs with more than 10 employees, ICT for smart working (communication and information sharing), two months later, the Italian lockdown of activities, was already acquired and therefore not suitable to make a difference in respondents’ thoughts.

Following suggestions received in our initial testing of the questionnaire, our study also considered the potential influence on resilience of incremental financial resources available in case of need. Other studies have pointed out that SMEs, are often undercapitalized compared with large companies, as banks and investment funds mostly cover the invested capital, being the “hidden majority shareholder” of firms. Supplementary financial resources alleviate the pressure on funding and allow the regular payment of current liabilities and debts in case of lack of inflows due unexpected economic shocks. This expected influence determined from the literature review has been also proven by our sample data.

The organizational resilience of SMEs is not separate from individual entrepreneurial resilience. As pointed out by scholars and confirmed by this study, an entrepreneur’s high personal resilience skills probably have a positive influence on the resilience of their SME. The entrepreneurial skills, which determine the success of an enterprise and define its entrepreneurial orientation and strategic choices, cannot but influence the organization’s ability to resist and recover. It is difficult to imagine the success of an army without its commander.

Our study also tested the impact on resilience of other demographic factors, such as the size of the business, the family nature, or the presence of woman directors on the BoD. Neither of the first two variables was statistically significant, and the third was only partially statistically significant. Regarding company size, resilience ability can be a delicate balance of flexibility, which is typical of the small size, and at the same time the possibility of having a more solid structure (large firms). The joint effect can be an insensitivity of resilience to size. A little more surprising is the absence of a statistically significant association between resilience and the family nature of the SME. The situation resulting from COVID-19 may have affected the two different groups similarly (family and non-family firms), or, more simply, the resilience capacity was not explainable by the family nature of the firm, as there were cases of high or low resilience in both contexts. Finally, the influence of presence in the BoD of woman directors was not only negative but also only moderately significant (*p* < 0.1), and only in one specification of the models. This result could have been influenced by a greater severity in evaluating its resilience by woman respondents, who were numerically very few.

Another issue that our study addressed was differences between resilience to the COVID-19 pandemic and the organizational (general) resilience of SMEs. Our research found no statistically relevant differences between the two resiliencies from respondent replies. Despite the magnitude of the effects of the COVID-19 pandemic, three months after the start of the pandemic, SMEs believed they could resist and recover similarly, because their ability remained stable or, more probably, because they evolved accordingly.

Based on this, the research hypotheses H1 and H2—the influence of (C1) control tools and supplementary finance (BC) on resilience to COVID-19—are confirmed. In contrast, H3 is only partially confirmed, since ICT tools were not significant in the analysis of Column 3 of Table 4. As to the existence of other variables influencing resilience to COVID-19 in our model, both family nature and company size did not show a statistically significant effect: Resilience is therefore not affected by these two factors. Therefore, H4 is not confirmed. The woman respondents of companies with women directors on the BoD may have been more cautious in answering about their ability to be resilient. Columns 4 and 5 confirm H5, that organizational (general) resilience and resilience to COVID-19 are similar, as the high correlation between the two values detected in the preliminary analysis phase suggested.

## Robustness test on the results

The models expressed in Table 4, Columns 3 and 5, were subjected to some robustness tests regarding the results. More particularly, in line with other studies, our models were also arranged in homogeneous subsets of data to consolidate the explanatory value of the results. Details of these integrative regressions are available on request. The first test concerned the group of family businesses (FAM = 1) and, that of non-family businesses (FAM = 0). The small (EMPL2019 < 50) and medium-sized enterprises (50 < EMPL2019 < 250) were analyzed separately, as well as manufacturing SMEs (NACE C macro-sector) against non-manufacturing ones (NACE F and G macro-sectors). The results are substantially in line with what has already been noted and what is represented in Table 4.

## Conclusions, limitations, and future developments

The magnitude and extension of the economic crisis derived from the COVID-19 pandemic has had dramatic effects on SMEs’ survival. Organizational resilience has been a relevant factor in supporting companies to react to and recover from adverse conditions. Management control systems, integrated information systems (ERP), and financial resources are three important elements of the material and resource enablers of resilience. As has been observed, amid the crisis, the managerial ability to make forecasts and anticipate management facts, the capacity to manage finance, the integration of business information into one system, and the availability of supplementary financial resources are what have allowed firms to survive.

This study investigated the organizational resilience of SMEs using data collected in 2020 from 143 companies in the provinces of Verona and Vicenza, a few months after the start of the COVID-19 pandemic. Multivariate OLS regressions were performed that identified some managerial and organizational factors that could affect the resilience of SMEs to the COVID-19 pandemic. As expected, the results of our regressions highlighted a positive influence on resilience of using management control tools, such as economic and treasury budgets or having a contingency plan. Supplementary financial resources had positive effects too. A high degree of resilience was also present in companies that declared a strong intention to use ERP systems. In contrast, three months after the start of the pandemic and the consequent lockdowns imposed by authorities, the use of ICT tools for smart working (remote communication and document sharing) did not reveal a statistically significant influence on resilience to the COVID-19 pandemic, as these were probably in use in all companies. In addition, our results showed that the resilience qualities of the entrepreneur exerted a positive influence, whereas the presence of women on the BoD had a slightly negative effect on SME resilience. Finally, we found that both the family nature of the business and the size (number of employees) had no statistically significant effects.

This study has some limitations in its statistical methodology, in the sample used, and in the period considered. They could be resolved with further extensions of the study, implementing a longer longitudinal analysis in different contexts and other countries, eventually repeating the survey in 2023.

Despite these limitations, we believe that this study makes a useful contribution to the literature on organizational resilience in its analysis of the influence of managerial and organizational factors, with interesting empirical implications. First, SMEs should invest more in management control systems and contingency plans, the use of which should not be limited to large companies. The financial and expertise efforts required to acquire them are within reach; it is now necessary to proceed in this direction. Second, considering the typical scarcity of financial resources in SMEs, they should create a buffer of funds that can be used in cases of crisis or financial constraint. Giving this timely consideration, with a solid economic reasoning, and negotiating with lenders and shareholders is within the reach of SMEs. Third, investments in ERP systems and, more broadly, digital transformation should be a priority for most SMEs, speeding up operations, enabling remote management, and moving toward a stronger integration of processes. Fourth, the resilience of the entrepreneur who oversees the enterprise is fundamental whether family or non-family businesses are being considered. Training programs to develop entrepreneurial skills and resilience are thus very important. Finally, men and women members of the BoD should each contribute to business resilience, whatever the size of the firm.

**Table 1:** Definition of variables (t0 = 1.1.2020, t1 = 1.5.2020, t2 = 1.7.2021)

Variable	Definition
R2	Resilience of the firm to COVID-19 (t1)
R22	Resilience of the firm to COVID-19 (t2)
R10	Resilience of the firm (t0)
R11	Resilience of the firm (t1)
R12	Resilience of the firm (t2)
C0	Use of economic and treasury budgets, presence of continuity plan (t0)
C1	Use of economic and treasury budgets, presence of continuity plan (t1)
C2	Use of economic and treasury budgets, presence of continuity plan (t2)
BC0	Availability of unused or supplementary financial resources on request (t0)
BC1	Availability of unused or supplementary financial resources on request (t1)
ERPI0	Intention to use ERP systems (t0)
ERPI1	Intention to use ERP systems (t1)
ERPI2	Intention to use ERP systems (t2)
ICTU0i	Use of ICT tools to communicate at a distance (t0)
ICTU1i	Use of ICT tools to communicate at a distance (t1)
ICTU2i	Use of ICT tools to communicate at a distance (t2)
G1	Resilience of the entrepreneur (t1)
G12	Resilience of the entrepreneur (t2)
G2	Presence of women on the Board of Directors (t1)
G22	Presence of women on the Board of Directors (t2)
FAM	Dummy variable containing 1 if family firm, 0 otherwise
lnEMPL2019	Natural logarithm of the number of employees as of 31.12.2019
lnEMPL2020	Natural logarithm of the number of employees as of 31.12.2020

**Table 2:** Descriptive statistics (143 observations)

Variable name	n.	Mean	Std. dev.	Min.	Max.
R2	143	5.16	1.06	2	7
R22	46	5.35	1.15	2.75	7
R10	143	5.20	1.18	1	7
R11	143	4.90	1.27	1	7
R12	46	5.34	1.09	1.75	7
C0	143	3.99	1.86	1	7
C1	143	4.04	1.85	1	7
C2	46	4.41	1.85	1	7
BC0	143	4.12	1.59	1	7
BC1	143	4.07	1.61	1	7
BC2	46	4.52	1.58	1	7
ERPI0	143	5.07	1.76	1	7
ERPI1	143	5.19	1.77	1	7
ERPI2	46	5.26	1.57	1	7
ICTU0i	143	3.73	2.09	1	7
ICTU1i	143	3.96	1.93	1	7
ICTU2i	46	4.72	2.15	1	7
G1	143	5.69	1.35	1	7
G12	46	5.80	1.36	1	7
G2	143	4.57	2.40	1	7
G22	46	4.63	2.36	1	7
FAM	143	0.69	0.46	0	1
lnEMPL2019	143	3.36	1.01	2.30	5.394
lnEMPL2020	46	3.65	0.83	2.30	5.203

**Table 3:** Pairwise correlation matrix (only significant correlations)

	(1)	(2)	(3)	(4)	(5)	(6)	(7)	(8)	(9)	(10)
(1) R2	1									
(2) R11	0.73	1								
(3) C1	0.45	0.36	1							
(4) BC1	0.35	0.35	0.19	1						
(5) ERPI1	0.28	0.27	0.28	0.18	1					
(6) ICTU1i	0.17		0.21			1				
(7) G1	0.41	0.39	0.38	0.35			1			
(8) G2								1		
(9) FAM								0.21	1	
(10) lnEMPL2019									-0.19	1

Note. 143 observations. Only correlations significant at least at 5% are reported

**Table 4:** OLS regressions - Results (robust standard errors)

	(1) R2,	(2) R2,	(3) R2,	(4) R11,	(5) R11,
Variables considered in the model	Only Control Var.	Control and Indep. Var.	All Var.	Control and Indep. Var.	All Var.
FAM	0.125	0.064	0.044	0.247	0.204
	(0.184)	(0.176)	(0.154)	(0.231)	(0.208)
lnEMPL2019	0.089	0.1000	-0.018	0.090	-0.043
	(0.089)	(0.082)	(0.076)	(0.099)	(0.100)
G1	-	0.336***	0.181**	0.384***	0.229***
		(0.059)	(0.069)	(0.066)	(0.081)
G2	-	-0.051	-0.0531*	-0.071*	-0.073*
		(0.035)	(0.032)	(0.540)	(0.040)
C1	-	-	0.156***		0.119**
			(0.046)		(0.060)
BC1	-	-	0.117**		0.146**
			(0.057)		(0.070)
ERPI1	-	-	0.104**		0.141**
			(0.051)		(0.512)
ICTU1i	-	-	0.053		0.060
			(0.044)		(0.051)
Constant	4.778***	3.097***	2.548***		1.887***
	(0.340)	(0.465)	(0.491)		(0.541)
R-squared	0.008	0.189	0.350	0.179	0.302
R-squared adj.	-0.006	0.166	0.311	0.155	0.260
VIF mean	1.04	1.07	1.20	1.07	1.20
F	0.68	9.34***	9.74***	9.81***	10.59***
Observations	143	143	143	143	143

Note. The dependent variable is alternatively R2 or R11. Robust standard errors in round parentheses;

*** p < 0.01, ** p < 0.05, * p < 0.1

## Electronic supplementary material

Below is the link to the electronic supplementary material.

Supplementary Material 1

## Data Availability

Not applicable.

## Appendix

Questionnaire submitted to companies – list of questions

(* – Responses with Likert scale 7: 1 – Completely disagree, 7 – Completely agree)

Q1. (R1A) My company is able to withstand adverse external conditions (resilience)*.

Q2. (R1B) My company is able to recover from adverse external conditions (resilience)*.

Q3. (R1C) My company is able to adapt strategies in relation to adverse external conditions (resilience)*.

Q4. (R1D) My company is able to reconfigure the business model in relation to adverse external conditions (resilience)*.

Q5. (R2A) My company is able to resist the COVID-19 pandemic*.

Q6. (R2B) My company is able to recover from the COVID-19 pandemic*.

Q7. (R2C) My company is able to reconfigure itself following the COVID-19 pandemic*.

Q8. (R2D) My company is able to adapt strategies following the COVID-19 pandemic*.

Q9. (ICTU) Smart working IT tools (ICT) should be intensively used*.

Q10. (ERPI) An integrated management system (ERP) should be intensively used in the company*.

Q11. (C.a) The company has a structured system of monthly economic budgets*.

Q12. (C.b) The company has a treasury budget on a monthly or fortnightly basis*.

Q13. (C.c) The company has a well-structured business continuity plan*.

Q14. (BC.a) The company has substantial unused credit lines*.

Q15. (BC.b) The shareholders are available to inject the liquidity necessary to guarantee business continuity*.

Q16. (G1) Those who predominantly govern the company (Entrepreneur, Chief Executive Officer, General Manager) have high personal resilience skills*.

Q17. (G2) In the top management (Entrepreneur, Chief Executive Officer, General Manager) there are woman members*.

Q18. (FAM) Family business: if one or two families directly or indirectly control the business and have at least one member on the BoD (Yes/No).

Q19. (EMPL2019) Employees at 31/12/2019.

## Declarations

### Data

The datasets generated and analysed during the current study are not publicly available due the fact that they constitute an excerpt of research in progress but are available from the corresponding author on reasonable request.
