# Supplementary material for: The role of management control and integrated information systems for the resilience of SMEs
Source: Rev Manag Sci. 2023 Mar 28:1–23. Online ahead of print. doi: 10.1007/s11846-023-00657-6 (PMC10044055; doi:10.1007/s11846-023-00657-6)
Supplement: Supplementary file 1 — Supplementary Material 1 [file 11846_2023_657_MOESM1_ESM.docx]

**Small and medium-sized enterprises resilient to COVID-19 pandemic: The role of management control and integrated information systems**

# Abstract

This paper investigates the resilience of small and medium-sized enterprises (SMEs) in relation to the COVID-19 pandemic and particularly the influence exerted by certain factors, such as the use of budgeting and business continuity plans, availability of supplementary capital, use of enterprise resource planning (ERP) systems, information and communication technologies (ICT) tools for communication and remote working, resilience of entrepreneurs, family business characteristics, and presence of women on the board of directors. For this purpose, in the late spring of 2020, a questionnaire was sent to limited liability SMEs in Verona and Vicenza provinces (Italy) operating in the manufacturing, construction and distribution sectors. Respondents were asked to respond to a set of questions and to evaluate the resilience of their firms as of 1 January 2020 and 1 May 2020. Using a multivariate regression model to analyse data from the 143 questionnaires received, we found a positive influence on both COVID-19 and general resilience of budgeting and business continuity tools, availability of supplementary financial resources, and resilience of the entrepreneur. SMEs with a high intention to use ERP systems also had high resilience. Despite the limitations concerning the sample and period considered, we believe that this study contributes to both the academic debate, by illustrating the influence of certain factors on the resilience of SMEs, and practice, by supporting a greater empirical use of control tools and ERP systems by SMEs.

**Keywords**: COVID-19, Resilience, SMEs, Resource-based view, Dynamic capabilities

**JEL**: L29, M10, M15, C29

# 1. Introduction

The recent COVID-19 pandemic has had a profound impact on the economic and social context of individuals as well as the operation of companies. In February 2020, within just a few days, Italian companies had to reorganize their internal processes, maximizing their digitalization in preparation for a progressive switch-off of in-person activities (Breier et al. 2020; De Massis and Rondi 2020). Some were equipped with highly integrated information systems, such as enterprise resource planning (ERP), others had only some parts in use (legacy systems) or did not have them at all. Many companies had no business continuity plan and had never simulated their operation in such an adverse business scenario. This situation has proved particularly critical in the context of small and medium-sized enterprises (SMEs), which constitute the backbone of Italian as well as global production and tend to have limited digitalization of administrative processes, limited adoption of ERP systems and low adoption of forecasting control tools (economic and financial budgets). Nonetheless, in recent decades, SMEs, which are universally recognized as having remarkable speed and agility, have also demonstrated extraordinary resilience, managing to resist and recover from crisis situations, by reconfiguring their business and adapting new strategies. Will they also be able to withstand the crisis following the COVID-19 pandemic? And if so, are there factors that can affect this resilience and the general resilience of the company?

To answer these research questions, a study based on a questionnaire sent to Veneto SMEs in the provinces of Verona and Vicenza was launched in the spring of 2020. To gather more public information as well as for relevance and homogeneity purposes, manufacturing, construction and distribution companies were selected. Chief financial officers or board of directors (BoD) members were asked about their resilience before (1 January 2020) and after (1 May 2020) the outbreak of the pandemic. We collected information regarding the use of budgets, contingency plans, ERPs, and available integrative capitals as well as some variables related to the personal resilience of the entrepreneur, the presence of women on the BoD, the family nature of the firm and its size. Responses were provided with a seven-point Likert scale. In total, 170 questionnaires were received from about 5,000 companies active in the area. From the analysis of the 143 valid questionnaires, we surprisingly found good resilience in the SMEs, both to COVID-19 and in general. After the factor analysis, we proceeded with a series of multiple regressions in which resilience was the outcome variable. The results highlighted a positive influence on resilience from using economic and treasury budgets or the presence of a contingency plan. Supplementary financial resources had positive effects too. A high degree of resilience was also present in companies that declared a high intention to use ERP systems. On the other hand, three months after the outbreak of the pandemic and the consequent lockdowns imposed by authorities, the use of information and communication technologies (ICT) tools for remote communication and sharing of documents had not revealed a statistically significant influence on resilience. In addition, our results showed that the resilience qualities of the entrepreneur exerted a positive influence, whereas the presence of women on the BoD had a slight negative effect on SME resilience. Finally, we found that both the family nature of the business and the size (number of employees) had no statistically significant effects.

This study has some limitations, including the limited sample size, the restricted geographic area and the short period considered, which suggest the need for its extension and repetition after some time. The statistical analysis also could be improved by studying alternative models or investigating moderation and mediation effects. Nonetheless, even with these limitations, we believe that this study contributes to the academic debate on SMEs’ resilience in the COVID-19 pandemic period. It also has practical implications for companies and practitioners by reaffirming the importance of control tools and financial buffers to foster SMEs’ resilience. We also endorse ERP implementation as the way to advance digital innovation in information systems.

# 2. Literature review

The issue of the resilience of businesses, particularly of SMEs, has been recurring since the 2000s, because of a series of increasingly frequent economic crises with ever stronger effects.

Briefly, resilience can be defined as the ability to resist and recover from adverse phenomena (Madni and Jackson 2009). It is a concept that has been used in various scientific fields, including engineering and technical fields (i.e. the ability of systems to remain functional during and after adverse situations), the organizational field (i.e. the ability of organizations to adopt behaviours capable of reducing the impact of these adverse phenomena), the social field (i.e. the ability of individuals and communities to reduce these negative effects) and the economic field, identified as the ability of businesses and economies to absorb negative effects caused by unexpected adverse events (Radovic-Markovic and Tomaš 2018).

The importance of the concept of resilience in the economic field is supported by the growing attention that supranational organizations—including, recently, the International Labour Organization and the Organisation for Economic Co-operation and Development—individual nations and their regional integrations, such as the European Union, are dedicating to the topic through specific reports and actions.

Promoting the resilience of businesses, and particularly of SMEs, is now a priority and a shared objective (Ates and Bititci 2011). After all, micro, small and medium-sized enterprises constitute the backbone of the Italian as well as the global economy, numerically representing in both cases over 90% of companies and employing more than 50% of the workforce (World Bank 2021). Of these businesses, approximately 70% are estimated to be family businesses (Zellweger 2017).

In economic studies, the resilience of businesses has been studied mostly in the entrepreneurship (De Vries and Shields 2006; Korber and McNaughton 2018) and family business literature, but some research has also focused on organizational and managerial effects of resilience (Skouloudis et al. 2020).

Business resilience has two different aspects: the first concerns being ready to identify and address potential negative effects and is thus a form of ex-ante resilience; the second is an ex-post form of reaction to adverse effects and adaptation to new contexts. When considering the latter aspect, the resilience of a company is a resource that the company owns (or does not) internally (Korber and McNaughton 2018). Consequently, there is a strong link between resilience and the resource-based view (RBV) and the concept of dynamic capability that could be considered his evolution. A business using the RBV approach identifies, among its sources of competitive advantage, the resources that have the traits of evaluability, uniqueness, imperfect imitability, and non-substitutability. This is a relatively static view because it does not provide an explanation of how resources evolve over time in changing environments. Conversely, the concept of dynamic capabilities focuses on the company’s ability to renew itself and reconfigure its resources in changing environments. These dynamic capabilities are therefore the ability of a company to create, extend and modify its basic resources (Helfat 2007) and are by their nature intrinsic and innate to the SME rather than acquired from the outside (Battisti and Deakins 2017).

Having said all this, in February 2020, the unexpected happened. Only a few companies had a business continuity plan in place that addressed the pandemic risk and its severe effects.

Within a few days (Carugati et al. 2020; Kraus et al. 2020), Italian companies—the first in the European continent—faced an unprecedented lockdown of their physical activities. In the following month of March, the World Health Organization declared COVID-19 a pandemic, which in 2020 would cause over 75,000 deaths in Italy and over 1.5 million worldwide. In addition to the impact on social and health systems, the COVID-19 emergency is one of the major events that companies have had to face in recent decades (Margherita and Heikkilä 2021).

Certainly, the actions of governments, including the financial support they provided, mitigated the most macroscopic effects, but the importance of being resilient to this crisis scenario inevitably emerged. The COVID-19 pandemic therefore generated a new test for business resilience.

The ability to resist and reconfigure by modifying, if necessary, strategies and organizational structure, leveraging control tools, financial resources and information systems infrastructure has been fundamental for the survival of companies. Other positive contributions derived from the ability of a company to accelerate its digital transformation (i.e. the transition from substantially paper-based processes to digital ones) supported by integrated information systems, such as ERP.

The choice to integrate and manage business data in a digital format is a strategic decision (Jenson and Johnson 1999) that affects all business processes. However, traditionally, SMEs have been rather sceptical of ERP adoption because of fears of the excessive rigidity of these systems, as well as the high cost of implementation in terms of the resources and skills required (Buonanno et al. 2005; Muscatello et al. 2003).

Nonetheless, it is in the SME context that the effects of these unforeseen adverse events can have the most serious consequences (Ates and Bititci 2011), which is what this study seeks to address, building on the existing literature on business resilience (Bhamra et al. 2011), and analysing the influence exercised by management control systems, finance, and other company-specific variables on SMEs’ resilience. In particular, our research question asks whether the presence of economic and treasury budgets or business contingency plans, availability of additional capital, adoption of ERP systems and ICT for smart working, presence of women on the BoD, company size, and resilience of the entrepreneur affect business resilience. Regarding the potential effect of entrepreneurial resilience, in the context of SMEs, which are often also family businesses, the link between the two subjects—the entrepreneur and the company—is so strong that inevitably a high resilience of the former has a driving effect on the latter (Ayala-Calvo and Manzano-García 2014; Branicki et al. 2018). This obviously does not exclude the positive contribution to business resilience provided by other individuals, such as collaborators and managers, especially in times of crisis (Van Der Vegt et al. 2015). Broadly speaking, the resilience of an organization is influenced by two fundamental aspects, namely, the vulnerability of the system to which it refers (in turn an expression of its sensitivity, responsiveness, and exposure to events) and its adaptive capacity (Bhamra et al. 2011). In practice, it will vary from case to case, depending not only on internal variables within the organization but also on external ones, and being more or less resilient also depends on the type of event considered. Indeed, the resilience of a given SME can be influenced by particular aspects related to ownership, governance, organizational size and structure, type of processes implemented, and so on.

From a theoretical point of view, the resilience of an SME derives from three ‘enablers’, namely, goods and resources, dynamic competitiveness, and learning and culture (Pal et al. 2014). Five components can be identified in the first category, namely, material resources (systems and management processes), finance, social systems, network relationships and intangible assets. The second one (i.e. dynamic competitiveness) includes flexibility, solidity, ability to network and redundancy. The last group includes the leadership style and decision-making process, sense of community and well-being of employees.

In comparison with general resilience, some specific features in the adverse situation resulting from the COVID-19 pandemic that have affected SMEs must be recognized. First of all, the rapid decisions that needed to be made were imposed by a sequence of events of particular speed and intensity. Second, unlike in the past, bans were placed on economic activity that limited or completely blocked normal in-person operations. Finally, these events had a systemic and generalized impact, affecting the whole economic system and modifying the monetary cycle as a domino effect of the restrictions.

The literature has offered some best practices for developing and maintaining high organizational resilience also from the change management perspective. These actions include the adoption of long-term planning measures, strengthening of control systems, provision of alternative scenarios, rapid decision-making and implementation of strategies, use of performance measurement tools, continuous collection of scenario and context information, and efficient communication (Ates and Bititci 2011).

In our opinion, in the context generated by the COVID-19 pandemic, some of these actions will have a greater impact on the resilience of SMEs, in particular the use of budgeting, adoption of ICT systems for remote communication and sharing of data, implementation of advanced integrated information systems (ERP), and availability of supplementary financial resources to deal with treasury crises.

The use of control tools such as economic and treasury budgets, requires continuous monitoring of company performance through analysing the trend of gross contribution margins and earnings before interest and taxes (EBIT). It can also require the analysis of short-term cash flows and highlighting their possible pressures on financial positions (Bruni 1990; Brusa and Dezzani 1983; Drury 2018). Accordingly, despite the growing instability and uncertainty of the past two decades (Ekholm and Wallin 2000), the use of budgets supports the resilience of SMEs. The same can be said for the adoption of a corporate business continuity plan (Alesi 2008) and ERP adoption, although it has also been found that this practice is still not widespread in SMEs (Bhamra et al. 2011; Lindström et al. 2010). A positive support to SMEs resilience also in times of pandemic could arise from investments in ICT (Gunasekaran et al. 2011; Carugati et al 2020).

With regard to the availability of supplementary financial resources (in case of need), this is a prescription judged positively in the literature (Pal et al. 2014), although it is sometimes difficult to activate in SMEs.

Another aspect to be considered is the family status of the company. Regardless of the difficulties in defining when a firm can be considered a family-type business, especially from an operational point of view (Roffia et al. 2021), it has already been noted that family businesses can be more resilient. According to some authors, this would be a characteristic feature because of the relationship with the family (Kachaner et al. 2012). The presence of further socio-emotional benefits brought by family members in addition to those of an economic nature should extend the survival of businesses in evident crisis situations beyond any reasonable economic considerations, effectively increasing their resilience (Gomez-Mejia et al. 2007).

Also worthy of consideration are the possible influences on resilience exercised by women holding top management positions and company size, regarding which, however, there are conflicting positions among scholars.

Therefore, the analysis in this study passes through an empirical verification of the validity of some factors in influencing the resilience of SMEs to the crisis originated by the COVID-19 pandemic and whether, eventually, they are also valid for the general resilience of SMEs.

In summary, we formulate the following hypotheses:

H1: The adoption of control tools such as budgets affects the resilience of SMEs to the COVID-19 pandemic.

H2: The availability of additional financial resources, activated if needed, strengthens this resilience.

H3: The adoption or the intention to adopt ERP-type information systems and ICT tools for smart working and remote communication promotes high resilience in SMEs.

H4: Other factors related to the context of SMEs, such as company size, resilience of the entrepreneur, family business type or presence of female members on the BoD, can have a significant influence on this resilience.

H5: Factors possibly influencing the resilience to COVID-19 are ‘common’ to the general resilience of businesses.

# 3 Research methodology

To answer the research questions, a study was carried out based on a questionnaire addressed to manufacturing, construction and distribution companies operating in the Veneto Region in the provinces of Verona and Vicenza with employees numbering between 10 and 249.

From the definition of SME adopted within the European Union, we decided to use only the requirement regarding employees, leaving out the simultaneous use of sales revenues and total balance sheet assets. Data collection started in May 2020, three months after the arrival of the COVID-19 pandemic in Italy. The research goal was the analysis of the resilience of SMEs both to COVID-19 and in general terms in the light of some potential influencing factors such as the use of management control tools, adoption of ERP systems and availability of additional capital in case of need.

In a preliminary phase, two members of the BoD were interviewed to gather some basic information regarding our topic (Woodside and Wilson 2003), which was useful for preparing an online questionnaire on an “internet form” through which to ask respondents to express themselves with a Likert scale from 1 to 7 (1: totally disagree, 7: completely agree) on some statements relating to the ability to resist and recover from adverse phenomena by changing and reconfiguring the business, use of economic or financial budgets, presence of a business continuity plan, availability of additional financial resources in case of need. Information on entrepreneur resilience was also collected.

Respondents were asked to refer in their responses to two particular time points, 1 January 2020 and 1 May 2020, ideally before the pandemic and two months after the introduction of restriction measures in Italy to mitigate the effects of COVID-19. A variable was associated with each statement, but the overall number of statements was limited to raise the response rate. At the end of the questionnaire, we inserted a section to collect some demographic information, such as the year of birth, NACE code (which is the standard European nomenclature of productive economic activities), number of employees and sales turnover. A complete list of the variables considered is given in the Appendix. About 5,000 SMEs fulfilling our requirements in terms of legal form, status (in business), geographical area and size were contacted via email out of a total of 5,421 companies listed by the Italian National Institute of Statistics (Istituto Nazionale di Statistica; ISTAT) in 2019. The invitation was addressed to the chief financial officer, to a member of the BoD or the majority shareholder (depending on our contacts) because they are the most qualified individuals to discuss control systems and integrated information systems, as well as the main decision-makers regarding their adoption. The questionnaire indicated the purpose of the research and provided guidance on how to respond. A total of 170 questionnaires was collected, but for homogeneity with respect to the purposes and our target companies, the analysis was limited to 146 cases, three of which were subsequently removed as outliers. The resilience of companies was investigated by asking four different questions to the interviewees, in order to understand its different facets. The survey was conducted in relation to both resilience to COVID-19 and general resilience. In light of the research questions and the literature review, we used specific variables to monitor the following issues in the sample of SMEs considered: (a) use of budgets as control tools and preparation of a business continuity plan and (b) availability of supplementary financial resources. Both can improve the resilience of a business by forecasting financial results and simulating how to guarantee continuity of action in crisis situations by preparing the financial resources required to overcome the financial stress deriving from the limitations to activities. In the same direction, we monitored (c) the intention to use ERP-type systems and the use of ICT for smart working. Given the traditional distrust of SMEs regarding the adoption of advanced digital infrastructures, which for many years has postponed their implementation—because of the unpredictability of contingent situations worsening that has caught many of them in midstream—we focused on the intention to use ERP-type systems instead of their actual use. As is generally known, ERPs allow for the integrated management of a company’s (digital) information system, making it possible to operate the company remotely. It was decided to identify the use of the second category of tools, ICT, because they also allow remote communication between different people and the exchange of documents and information. To increase the reliability of the answers, this variable was initially identified in the opposite direction (as NOT used) and then reversed for the subsequent analysis. Finally, we decided to analyse the degree of resilience of the entrepreneur (in addition to that of the SME), the degree of participation in decisions of the BoD by female members and some context variables such as the ‘family’ nature of the company and its size in terms of number of employees. Regarding the first context variable, a firm was considered a family business when the family had ownership control and at least one member active on the BoD (Roffia et al. 2021). Regarding the second variable, it was decided to use the natural logarithm of the number of employees as a measure of company size.

Before defining the model and carrying out the first descriptive statistics, we conducted exploratory factor analysis on the variables relating to the resilience to COVID-19, to general resilience, to control tools and business continuity plans, and to availability of additional capital. Through this step, it was possible to reduce the analysis of the four aspects mentioned above to four factors/variables, naming them respectively R2 (COVID-19 resilience), R1 (general resilience), C (controls) and BC (availability of capital). Given that for the latter three (R1, C and BC) the identification was performed in respect of both time *t*_0_ and time *t*_1_, the final data set encoded these factor data as R10, R11, C0, C1, BC0 and BC1.

The analysis used to answer the research questions is based on multivariate ordinary least squares (OLS) regressions where the dependent variable is resilience, and the explanatory variables are those previously mentioned and reported in Table 1. In turn, resilience was considered in a dual way, namely, to COVID-19 and in general and wide terms; therefore, two separate regression models were used for both the first type of resilience and the second one.

[Insert Table 1 here]

# 4 Data analysis and discussion of the results

Table 2 shows the descriptive statistics of the variables considered in this study in our sample of SMEs, including minimum, maximum, mean and standard deviation.

[Insert Table 2 here]

The data referring to May 2020 (*t*_1_), in Italy after two months of lockdown of the activities in presence, showed a more than fair capacity of resilience declared by the companies both to COVID-19 (R2 = 5.2) and in general (R11 = 4.9), even if resilience to the latter was decreasing (*p* < 0.01) from the beginning of the year (R10 = 5.2) and had a slightly higher standard deviation (1.3 against the previous 1.2).

Factor C1, which includes variables relating to the use of an economic budget, the use of the treasury budget and the preparation of a business continuity plan, as well as factor BC1, which represents the availability of supplementary capital, had good values (4.0 and 4.1 respectively). It is worth noting that we found a greater variability of the data expressed by a higher standard deviation, respectively equal to 1.9 and 1.6 (always on a seven-point Likert scale). Surprisingly, the COVID-19 pandemic, compared with the situation at 1 January 2020, has not substantially changed the adoption of control tools (*p* < 0.01), whereas the availability of additional financial resources has slightly decreased (*p* < 0.01). Much more evident, however, is the difference between 1.1.2021 and 1.5.2021 for the intention to use ERP systems and the actual use of ICT tools for remote communication. They went from 5.08 to 5.19 and from 3.76 to 3.97 respectively (*p* < 0.01) without particular changes in the standard deviation. Approximately 70% of the companies declared that they are family firms, according to the previously defined criteria. This is not very different from data found in other studies for the Italian and European context (Roffia et al. 2021). The size of the company in terms of employees fluctuated between 1 and 220 employees (ln 220 = 5.394).

Table 3 shows the correlation matrix between the variables at time *t*_1_ used in the linear regressions referred to in Table 4. The significant correlations (*p* < 0.05) show some associations between the two resiliencies R2 and R11 and the variables previously illustrated without, however, exceeding values of 0.4 (the correlation between R2 and R11 is also reported but it is not useful for our analysis, since they are alternative outcome variables in our models). Table 4 reports the regression analyses carried out regarding resilience R2 (to COVID-19) and R11 (general) as output variables. The analysis proceeded in successive steps to insert additional explanatory variables in the model to increase the goodness of fit of the model expressed by the *R*^2^ value and the associated *F*-test. Therefore, in Column 1, only the control variables FAM and lnEMP2019 have been inserted; Column 2 also includes the independent variables related to ERPI and ICTUi as well as G1 and G2; finally, in Column 3 C1 and BC1 are also added in regression. Passing from the first to the third column, a progressive increase in *R*^2^ can be found (from 0.01 to 0.35), with gradually increasing *F*-tests (in the third column the value is close to 10, *p* < 0.001). The adjusted *R*^2^ value is substantially in line with the above, while adjusting the number of regressors considered. The post-regression checks verified the existence of the conditions for the validity of the model and, in particular, the absence of heteroskedasticity, collinearity (variance inflation factor) and the correct formulation of the model. In observing the results of Column 3, the variables that were found to be significant and therefore influencing the resilience to COVID-19 of SMEs were the use of control tools (budgets and contingency plans) C1 (+), availability of incremental finance BC1 (+), intention to use ERP ERPI1 (+) and resilience of the entrepreneur G1 (+). All four variables revealed a positive influence on resilience to COVID-19. Slightly significant (*p* < 0.1), but with a negative sign, was the influence of the presence of female members on the BoD G2 (-). The remaining variables were not significant except for the constant of the model. Among them was also the use of ICT tools (ICTU1i), which was therefore unable to affect the levels of resilience, perhaps also because by the time *t*_1_ to which the analysis refers, companies had already adopted these tools in a generalized way. It should also be noted that the actual use of ERP tools, already from the preliminary phase of the correlation analysis, was not significant and therefore discarded in our analyses. Based on the above, the research hypotheses H1 and Hp2 (the influence of C1 control tools and supplementary finance (BC) on resilience to COVID-19) are confirmed. On the other hand, H3 has only partial acceptance, since the ICT tools were not significant in the analyses of Columns 1 to 3. As to the existence of other variables influencing the resilience to COVID-19 in our model, both the family nature and the company size did not show a statistically significant effect: resilience is therefore not affected by these two factors. Therefore, H4 has a negative answer. Columns 4 and 5 retrace the previous analysis on the general resilience of SMEs. The results are similar to those relating to resilience to effects of the COVID-19 pandemic, confirming H5 (that resilience to COVID-19 and general resilience of SMEs are similar to each other), as the high correlation between the two values detected in the preliminary analysis phase suggested.

# 5 Robustness test on the results

The models expressed in Table 4, Columns 3 and 5 were subjected to some robustness tests regarding the results. More particularly, in line with other studies, to consolidate the explanatory value of the results, our models were were also sought in homogeneous sub-sets of data. Details of these integrative regressions are available on request. The first test concerned the group of family businesses and by difference also that of non-family businesses (FAM =1). The small (EMPL2019 < 50) and medium-sized enterprises (50 < EMPL2019 < 250) were analysed separately, as were manufacturing SMEs (ISTAT C sector). The results are substantially in line with what has already been noted above and represented in Table 4.

# 6 Conclusions, limitations, future developments

This study investigated the resilience of SMEs using data from 143 companies in the provinces of Verona and Vicenza collected a few months after the outbreak of the COVID-19 pandemic. Multivariate OLS regressions were performed that identified some factors that could affect the resilience of responding SMEs. Management control tools, such as economic and treasury budgets plus contingency plans, availability of additional capital and intention to adopt ERP, influence the resilience to the COVID-19 pandemic as well as general resilience of firms. The same applies to the personal resilience of the entrepreneur, whereas, the family nature and size of the company have no significant effect. The influence of women on the BoD is moderately negative. This study certainly has some limitations in the statistical methodology, in the sample used and, in the period considered. Nevertheless, despite such limitations, we believe that it is a useful contribution to studies relating to the resilience of businesses to COVID-19, with interesting empirical implications. First, SMEs should invest more in management control systems and contingency plans, whose use should not be limited to large companies. Second, in light of the typical scarcity of financial resources in SMEs, they should better manage financial risks related to both debts and equity capitals. Third, digital transformation and investments in ERP systems should be a priority for most of them, speeding up operations, enabling remote management and moving towards a stronger integration of value chain elements. Fourth, the resilience of the entrepreneur is fundamental whenever family or non-family business are considered. Finally, male or female members of the BoD should both contribute to business resilience, whatever the size of the firm.

**Table 1.** Definition of variables (t0=1.1.2020, t1=1.5.2020)

| **Variable** | **Definition** |
| --- | --- |
| R2 | Resilience of the firm to COVID-19 |
| R10 | Resilience of the firm (t0) |
| R11 | Resilience of the firm (t1) |
| C1 | Use of economic and treasury budgets, presence of continuity plan (t1) |
| BC1 | Availability of unused or supplementary financial resources on request (t1) |
| ERPI1 | Intention to use ERP systems (t1) |
| ICTU1i | Use of ICT tools to communicate at a distance (t1) |
| G1 | Resilience of the entrepreneur |
| G2 | Presence of women on the Board of Directors |
| FAM | Dummy variable containing 1 if family firm, 0 otherwise |
| lnEMPL2019 | Natural logarithm of the number of employees as of 31.12.2019 |

**Table 2.** Descriptive statistics (143 observations)

| **Variable name** | **Mean** | **Std. dev.** | **Min.** | **Max.** |
| --- | --- | --- | --- | --- |
| R2 | 5.162 | 1.056 | 2 | 7 |
| R10 | 5.204 | 1.184 | 1 | 7 |
| R11 | 4.905 | 1.270 | 1 | 7 |
| C0 | 3.990 | 1.856 | 1 | 7 |
| C1 | 4.042 | 1.846 | 1 | 7 |
| BC0 | 4.119 | 1.589 | 1 | 7 |
| BC1 | 4.070 | 1.606 | 1 | 7 |
| ERPI0 | 5.070 | 1.759 | 1 | 7 |
| ERPI1 | 5.188 | 1.768 | 1 | 7 |
| ICTU0i | 3.734 | 2.086 | 1 | 7 |
| ICTU1i | 3.958 | 1.931 | 1 | 7 |
| G1 | 5.692 | 1.349 | 1 | 7 |
| G2 | 4.566 | 2.402 | 1 | 7 |
| FAM | 0.692 | 0.463 | 0 | 1 |
| lnEMPL2019 | 3.364 | 1.011 | 0 | 5.394 |

**Table 3.** Pairwise correlation matrix (only significant correlations)

|  | (1) | (2) | (3) | (4) | (5) | (6) | (7) | (8) | (9) | (10) |
| --- | --- | --- | --- | --- | --- | --- | --- | --- | --- | --- |
|  |  |  |  |  |  |  |  |  |  |  |
| (1) R2 | 1 |  |  |  |  |  |  |  |  |  |
| (2) R11 | 0.7329 | 1 |  |  |  |  |  |  |  |  |
| (3) C1 | 0.4506 | 0.3570 | 1 |  |  |  |  |  |  |  |
| (4) BC1 | 0.3513 | 0.3541 | 0.1902 | 1 |  |  |  |  |  |  |
| (5) ERPI1 | 0.2842 | 0.2728 | 0.2759 | 0.1788 | 1 |  |  |  |  |  |
| (6) ICTU1i | 0.1673 |  | 0.2144 |  |  | 1 |  |  |  |  |
| (7) G1 | 0.4122 | 0.3947 | 0.3803 | 0.3529 |  |  | 1 |  |  |  |
| (8) G2 |  |  |  |  |  |  |  | 1 |  |  |
| (9) FAM |  |  |  |  |  |  |  | 0.2084 | 1 |  |
| (10) lnEMPL2019 |  |  |  |  |  |  |  |  | -0.1939 | 1 |
|  |  |  |  |  |  |  |  |  |  |  |

*Note.* 143 observations. Only correlations significant at least at 5% are reported.

**Table 4.** Results (robust standard errors)

|  | (1) R2, | (2) R2, | (3) R2, | (4) R11, | (5) R11, |
| --- | --- | --- | --- | --- | --- |
| Variables considered | Only Control | Control and | All | Control and | All |
| Model | Var. | Indep. Var. | Var. | Indep. Var. | Var. |
|  |  |  |  |  |  |
| FAM | 0.1247 | 0.0639 | 0.0440 | 0.2470 | 0.2044 |
|  | (0.184) | (0.176) | (0.154) | (0.231) | (0.208) |
| lnEMPL2019 | 0.0887 | 0.1000 | -0.0183 | 0.0903 | -0.0426 |
|  | (0.0892) | (0.082) | (0.076) | (0.099) | (0.100) |
| G1 | - | 0.3364*** | 0.1807** | 0.3837*** | 0.2287*** |
|  |  | (0.059) | (0.069) | (0.0659) | (0.081) |
| G2 | - | -0.0513 | -0.0531* | -0.071* | -0.073* |
|  |  | (0.035) | (0.032) | (0.540) | (0.040) |
| C1 | - | - | 0.1559*** |  | 0.1190** |
|  |  |  | (0.046) |  | (0.060) |
| BC1 | - | - | 0.1175** |  | 0.1459** |
|  |  |  | (0.057) |  | (0.070) |
| ERPI1 | - | - | 0.1037** |  | 0.1413** |
|  |  |  | (0.051) |  | (0.512) |
| ICTU1i | - | - | 0.0539 |  | 0.060 |
|  |  |  | (0.044) |  | (0.051) |
| Constant | 4.778*** | 3.0974*** | 2.5480*** |  | 1.8874*** |
|  | (0.340) | (0.465) | (0.491) |  | (0.541) |
|  |  |  |  |  |  |
| R-squared | 0.0084 | 0.1895 | 0.3502 | 0.1786 | 0.3020 |
| R-squared adj. | -0.006 | 0.166 | 0.311 | 0.155 | 0.260 |
| VIF mean | 1.04 | 1.07 | 1.20 | 1.07 | 1.20 |
| F | 0.68 | 9.34*** | 9.74*** | 9.81*** | 10.59*** |
| Observations | 143 | 143 | 143 | 143 | 143 |

*Note.* The dependent variable is alternatively R2 or R11. Standard errors in round parentheses; p–values in parentheses.

*** p<0.01, ** p<0.05, * p<0.1.

# Declarations”

**• Competing interests:**

**• Data Availability Statement:**

# References

Ayala-Calvo, J. C., & Manzano-García, G. (2014). The resilience of the entrepreneur. Influence on the success of the business. A longitudinal analysis. *Journal of Economic Psychology, 42*, 126-135.

Alesi, P. (2008). Building enterprise-wide resilience by integrating business continuity capability into day-to-day business culture and technology. *Journal of business continuity & emergency planning, 2*(3), 214-20.

Ates, A., & Bititci, U. (2011). Change process: a key enabler for building resilient SMEs. *International Journal of Production Research, 49*(18), 5601-5618.

Battisti, M., & Deakins, D. (2017). The relationship between dynamic capabilities, the firm’s resource base and performance in a post-disaster environment. *International Small Business Journal, 35*(1), 78-98.

Bhamra, R., Dani, S., & Burnard, K. (2011). Resilience: the concept, a literature review and future directions. *International Journal of Production Research, 49*, 5375-5393.

Branicki, L.J., Sullivan-Taylor, B. and Livschitz, S.R. (2018). How entrepreneurial resilience generates resilient SMEs, *International Journal of Entrepreneurial Behavior & Research*, 24(7), 1244-1263.

Ekholm, B., & Wallin, J. (2000). Is the annual budget really dead? *European Accounting Review, 9*(4), 519-539.

Breier, M., Kallmuenzer, A., Clauss, T., Gast, J., Kraus, S., & Tiberius, V. (2020). The role of business model innovation in the hospitality industry during the COVID-19 crisis. *International Journal of Hospitality Management, 92*.

Bruni, G. (1990). *Contabilità per l’alta direzione.* Milano, EGEA.

Brusa, L., & Dezzani, F. (1983). *Budget e controllo di gestione.* Milano, Giuffrè.

Buonanno, G., Faverio, P., Pigni, F., Ravarini, A., Sciuto, D., & Tagliavini M. (2005). Factors affecting ERP system adoption: A comparative analysis between SMEs and large companies *Journal of Enterprise Information Management, 18*(4), 384-426.

Carugati, A., Mola, L., Plé, L., Lauwers, M., & Giangreco, A. (2020). Exploitation and exploration of IT in times of pandemic: from dealing with emergency to institutionalising crisis practices. *European Journal of Information Systems, 29*(6), 762-777.

De Massis, A., & Rondi, E. (2020). Covid-19 and the Future of Family Business Research. *Journal of Management Studies, 57*(8), 1727-1731.

De Vries, H., & Shields, M. (2006). Towards a theory of entrepreneurial resilience: a case study analysis of New Zealand SME owner operators. *Applied Research Journal, 6*, 33-43.

Drury, C. (2018). *Management and cost accounting.* Andover, Cengage.

Gomez-Mejia, L. R., Takács Haynes, K., Núñez-Nickel, M., Jacobson, K. J. L., & Moyano-Fuentes, J. (2007). Socioemotional Wealth and Business Risks in Family-controlled Firms: Evidence from Spanish Olive Oil Mills. *Administrative Science Quarterly, 52*(1), 106-137.

Gunasekaran, A., Rai, B. K., & Griffin, M. (2011). Resilience and competitiveness of small and medium size enterprises: an empirical research, *International Journal of Production Research*, 49(18), 5489-5509.

Lindström, J., Samuelsson, S., & Hägerfors, A. (2010). Business continuity planning methodology. *Disaster Prevention and Management, 19*(2), 243-255.

Jenson, R. L., & Johnson, I. R. (1999). The enterprise resource planning system as a strategic solution. *Information Strategy:* *The Executive's Journal, 15*(4), 28‐33.

Kachaner, N., Stalk, G., & Bloch, A. (2012). What you can learn from family business: focus on resilience, not short-term performance. *Harvard Business Review, 90*, 102-106.

Korber, S., & McNaughton, R. B. (2018). Resilience and entrepreneurship: a systematic literature review. *International Journal of Entrepreneurial Behavior and Research, 24*(7), 1129-1154.

Kraus, S., Clauss, T., Breier, M., Gast, J., Zardini, A., & Tiberius, V. (2020). The economics of COVID-19 initial empirical evidence on how family firms in five European countries cope with the corona crisis. *International Journal of Entrepreneurial Behavior & Research, 26*(5), 1067-1092.

Madni, A. M., & Jackson, S. (2009). Towards a conceptual framework for resilience engineering. *IEEE Systems Journal, 3*, 181–191.

Marchi, L. (2003). *I sistemi informativi aziendali.* Milano, Giuffrè.

Margherita, A., & HeikkilÄ, M. (2021). Business Continuity in the COVID-19 Emergency: A Framework of Actions Undertaken by World-Leading Companies. *Business horizons, 64*(5), 683-695.

Muscatello, J. R., Small, M. H. & Chen, I. J. (2003). Implementing enterprise resource planning (ERP) systems in small and midsize manufacturing firms. *International Journal of Operations & Production Management, 23*(8), 850-871.

Pal, R., Torstensson, H., & Mattila, H. (2014). Antecedents of organizational resilience in economic crises—an empirical study of Swedish textile and clothing SMEs. *International Journal of Production Economics, 147*, 410-428.

Radovic-Markovic, M., & Tomaš, R. (2018). Resilience of Small and Medium-Sized Enterprises in Terms of Globalization: An Evidence of Serbia. *International Journal of Entrepreneurship, 22*(3), 1-7.

Roffia, P., Moracchiato, S., Liguori, E., & Kraus, S. (2021). Operationally defining family SMEs: a critical review. *Journal of Small Business and Enterprise Development, 28*(2), 229-260.

Skouloudis, A., Tsalis, T., Nikolaou, I., Evangelinos, K., Leal Filho, W. (2020), Small & Medium-Sized Enterprises, Organizational Resilience Capacity and Flash Floods: Insights from a Literature Review. Sustainability. MDPI, 12, 7437.

Van Der Vegt, G. S., Essens, P., Wahlström M., & George G. (2015). Managing risk and resilience. *Academy of Management Journal, 58*, 971- 980.

Woodside, A. G., & Wilson, E. J. (2003). Case study research methods for theory building. *Journal of Business & Industrial Marketing, 18*(6/7), 493-508.

World Bank (2021). Small and medium enterprises (SMES) finance. Available at: https://www.worldbank.org/en/topic/smefinance. Accessed: September 15, 2021.

Zellweger, T. (2017). *Managing the Family Business: Theory and Practice.* Edward Elgar Publishing.

# Appendix

**Questionnaire submitted to companies – List of questions**

(* – Responses with Likert scale 7: 1 – Completely disagree, 7 – Completely agree)

Q1. (R1A) My company is able to withstand adverse external conditions (resilience)*.

Q2. (R1B) My company is able to recover from adverse external conditions (resilience)*.

Q3. (R1C) My company is able to adapt strategies in relation to adverse external conditions (resilience)*.

Q4. (R1D) My company is able to reconfigure the business model in relation to adverse external conditions (resilience)*.

Q5. (R2A) My company is able to resist the COVID-19 pandemic*.

Q6. (R2B) My company is able to recover from the COVID-19 pandemic*.

Q7. (R2C) My company is able to reconfigure itself following the COVID-19 pandemic*.

Q8. (R2D) My company is able to adapt strategies following the COVID-19 pandemic*.

Q9. (ICTU) Smart working IT tools (ICT) should be intensively used*.

Q10. (ERPI) An integrated management system (ERP) should be intensively used in the company*.

Q11. (C.a) The company has a structured system of monthly economic budgets*.

Q12. (C.b) The company has a treasury budget on a monthly or fortnightly basis*.

Q13. (C.c) The company has a well-structured business continuity plan*.

Q14. (BC.a) The company has substantial unused credit lines*.

Q15. (BC.b) The shareholders are available to inject the liquidity necessary to guarantee business continuity*.

Q16. (G1) Those who predominantly govern the company (Entrepreneur, Chief Executive Officer, General Manager) have high personal resilience skills*.

Q17. (G2) In the top management (Entrepreneur, Chief Executive Officer, General Manager) there are female members*.

Q18. (FAM) Family business: if one or two families directly or indirectly control the business and have at least one member on the BoD (Yes/No).

Q19. (EMPL2019) Employees at 31/12/2019.
